# Advances in Atrial Fibrillation Management: A Guide for General Internists

**DOI:** 10.3390/jcm13247846

**Published:** 2024-12-23

**Authors:** Hoang Nhat Pham, Ramzi Ibrahim, Hong Hieu Truong, Enkhtsogt Sainbayar, Viet Nghi Tran, Mahmoud Abdelnabi, Christopher Kanaan, Aadhavi Sridharan

**Affiliations:** 1 Department of Medicine, University of Arizona, Tucson, AZ 85719, USA; npham917@arizona.edu (H.N.P.); sainbayar@arizona.edu (E.S.); 2 Department of Cardiovascular Medicine, Mayo Clinic, Phoenix, AZ 85054, USA; ibrahim.ramzi@mayo.edu (R.I.); abdelnabi.mahmoud@mayo.edu (M.A.); kanaan.christopher@mayo.edu (C.K.); 3 Department of Medicine, Ascension St Francis, Evanston, IL 60202, USA; hhieu.truong@gmail.com; 4 Department of Medicine, Weiss Memorial Hospital, Chicago, IL 60640, USA; tranvietnghi2007@gmail.com; 5 Sarver Heart Center, University of Arizona, Tucson, AZ 85719, USA

**Keywords:** atrial fibrillation, management, guidelines, lifestyle modification, risk factor modification

## Abstract

Atrial fibrillation (AF) is the most common sustained cardiac arrhythmia, impacting approximately 6.1 million adults in the United States, with projections to increase two-fold by 2030. AF significantly increases the risk of stroke and other adverse cardiovascular events, leading to increased morbidity and mortality. The 2023 ACC/AHA/ACCP/HRS guidelines present a paradigm shift in AF management, moving from a duration-based classification to a more comprehensive, patient-centered approach. This includes a novel AF classification system that emphasizes early detection and intervention, including risk factors and lifestyle modification tailored to each patient’s risk profile. Moreover, the recommendations advocate for a multidisciplinary care model, ensuring coordinated management involving primary care providers and specialists. Primary care providers play a crucial role in initiating risk factor management and lifestyle interventions, even before the development of AF. This review aims to thoroughly examine the guidelines for the diagnosis and management of AF and equip general internists with the necessary insights to navigate the evolving landscape of AF care effectively.

## 1. Introduction

Atrial fibrillation (AF), the most common sustained cardiac arrhythmia, is characterized by abnormal electrical activity in the atrium with an irregular ventricular response [1]. The global prevalence of AF is on the rise, with an estimated 50 million cases worldwide in 2020. In the United States, approximately 6.1 million adults are affected, and this number is expected to double by 2030 [2,3,4]. AF significantly raises morbidity and mortality, imposing a major public health burden [5,6]. AF is the leading cardiac cause of stroke, with a five-fold increased risk due to its irregular rhythm with subsequent turbulent blood flow and higher likelihood of clot formation [7,8]. Additionally, AF is associated with an increased risk for adverse cardiac events, including heart failure (HF), myocardial infarction (MI), cognitive decline and dementia, and higher hospitalization rates and healthcare costs [9]. AF management often requires anticoagulation therapy to prevent thromboembolic events, though it carries a risk of bleeding complications. These risks, alongside other adverse cardiovascular outcomes, emphasize the need for early detection, proper management, and ongoing research to reduce its potential complications [10]. This review aims to thoroughly examine the newest 2023 ACC/AHA/ACCP/HRS guidelines for the diagnosis and management of AF, with a focus on key updates, including new AF classification, lifestyle and risk factor modification (LRFM), and early rhythm control and catheter ablation, to help general internists improve patient outcomes and adapt to the evolving landscape of AF care [4].

### Clinical Significance

- The 2023 ACC/AHA/ACCP/HRS guidelines for the diagnosis and management of AF emphasize a patient-centered approach, focusing on early detection and tailored lifestyle interventions.
- A multidisciplinary care model is promoted, enabling primary care providers to effectively manage risk factors and reduce morbidity and mortality associated with AF.

## 2. Atrial Fibrillation Classification

The 2023 guidelines advocate for the shift from the traditional duration-based classification of AF to a more holistic, patient-centered approach that emphasizes prevention through risk factor management and lifestyle changes throughout a patient’s life [4]. The introduction of AF stages addresses the limitations of the previous classification, which primarily focused on therapeutic interventions for diagnosed AF. The new stages recognize AF as a progressive disease that requires tailored strategies at each stage [4]. This approach emphasizes the importance of early interventions starting from Stage 1 (at risk for AF) by focusing on prevention and managing AF risk factors. Stage 2 (pre-AF) involves timely screening for high-risk patients, such as those with atrial enlargement, frequent atrial ectopy, atrial flutter, or short bursts of atrial tachycardia. Stages 3 and 4 correspond to diagnosed AF and permanent AF, respectively, and involve addressing pathophysiological changes, treating symptoms, and preventing complications. All stages are interconnected, with an emphasis on risk factor management across all stages [4].

The foundation for optimal management lies in addressing risk factors, comorbidity management, and promoting lifestyle changes to prevent AF using a comprehensive screening approach (HEAD 2 TOES: Heart failure, Exercise, Arterial hypertension, Diabetes, Tobacco, Obesity, Ethanol, Sleep) [4,11,12]. Once AF is diagnosed, three critical processes should be prioritized: **S**troke risk assessment and treatment, **O**ptimization of risk factors, and tailored **S**ymptom management strategies (**SOS**). Ultimately, ensuring Access to All Aspects of care for All (4 As) is essential for achieving meaningful improvements in AF management [4]. There are currently no studies validating the efficacy of the SOS algorithm given its recent introduction. However, the SOS algorithm shares several similarities with the existing ABC pathway (Atrial Fibrillation Better Care), which is based on three key pillars: “A” for avoiding stroke, “B” for better symptom control, and “C” for managing cardiovascular risk factors and other comorbidities [13]. The ABC pathway has been demonstrated to effectively reduce the risk of adverse clinical outcomes, overall mortality, and cardiovascular death, further reinforcing the rationale for the SOS algorithm [14,15,16].

## 3. Atrial Fibrillation Management Approach

Patients with AF often have multiple comorbidities, making a comprehensive, individualized approach essential. This approach should include guideline-directed LRFM, targeted interventions for AF symptoms, and strategies to mitigate the risk of stroke and other associated medical conditions. Such a holistic strategy aims to reduce the burden, progression, and complications of AF [4,17].

### 3.1. Lifestyle Intervention and Risk Factor Modification

With the updated AF classification, the new guideline emphasizes a more comprehensive management approach, focusing on lifestyle and risk factor modification (Table 1). This includes both primary and secondary prevention to reduce the onset, progression, and adverse outcomes of AF. Key targets for LRFM include obesity, physical inactivity, unhealthy alcohol consumption, smoking, diabetes, hypertension, and substance abuse.

Obesity results in direct atrial myocardium remodeling, creating a substrate for AF development and progression [18]. A 2015 meta-analysis of 51 studies involving 626,603 patients reported a 10% and 13% greater risk of postoperative and post-ablation AF for every 5-unit increased body mass index (BMI), respectively [19]. In the 2013 Abed trial, weight reduction as part of LRFM significantly decreased AF symptoms, recurrence, and burden in overweight and obese patients, with BMI ≥ 27 [20]. The 2015 LEGACY study, including 1415 AF patients with a median BMI of 33.6, found that losing ≥10% of body weight led to a six-fold increase in arrhythmia-free survival. However, weight fluctuations greater than 5% partially diminished this benefit, doubling the risk of AF recurrence [21]. The REVERSE-AF study, a subgroup analysis of LEGACY, further demonstrated the reversion of the type and natural progression of AF with weight loss in a dose-dependent manner [22]. Similarly, the SORT-AF trial reported a significant correlation between BMI and post-ablation AF recurrence for patients with persistent AF compared to paroxysmal AF patients (OR 1.154) [23]. In patients who are morbidly obese (BMI ≥ 40 kg/m^2^ or ≥35 kg/m^2^ with obesity-related complications), bariatric surgery prior to AF ablation was associated with a lower post-ablation AF recurrence (adjusted hazard ratio of 0.14) and, hence, can be considered in this patient population prior to ablation [24]. However, evidence for AF prevention through dietary supplements remains inconsistent [4].

Observational studies have shown an elevated risk of incident AF with a sedentary lifestyle [25,26,27,28]. Aerobic exercise interventions have been effective in reducing the burden of non-permanent AF while improving functional capacity, as measured by the 6-minute walk test (6 MWT), and enhancing the quality of life in both patients with permanent and non-permanent AF [29,30,31]. The 2023 ACTIVE-AF randomized clinical trial demonstrated that patients with paroxysmal or persistent symptomatic AF experienced fewer and shorter AF episodes, reduced symptom burden, and improved cardiorespiratory fitness when moderate-to-vigorous exercise (targeting 210 min per week) was supplemented to their usual care [32]. Nevertheless, caution is needed, particularly regarding adequate ventricular rate control and the potential for atrial myopathy from excessive exercise. Conversely, high-volume, high-intensity endurance training (≥3 h/day), especially in young male athletes, has also been associated with a higher incidence of AF [33,34,35,36]. The proposed mechanisms include atrial dilation, adrenergic and vagal activation, chronic inflammation, pulmonary foci, and interstitial fibrosis as a result of excessive strain through augmented cardiac output and atrial stretching [37].

Smoking and excessive alcohol consumption both adversely affect AF outcomes. Smoking is associated with an increased risk for post-ablation AF recurrence and worse cardiovascular outcomes in patients with AF, including stroke, HF, hospitalization, and death, and those who undergo tobacco cessation showed lower trends of incident AF and improved CVD outcomes [38,39,40]. Additionally, smoking reduces the efficacy of anticoagulation, thereby increasing stroke risk [41]. Therefore, smoking cessation through behavioral interventions and pharmacotherapy is strongly recommended [42,43]. Similarly, alcohol reduction to ≤3 standard drinks per week or complete abstinence is associated with a reduction in AF symptoms, burden, and progression because of the high risk of incident AF with alcohol consumption [12,20,21,22,44]. Conversely, there is limited evidence linking caffeine intake to an increased risk of AF, despite it frequently being reported as a trigger or an exacerbating factor for AF symptoms. In fact, moderate caffeine consumption has been shown to reduce AF incidence [45,46]. Therefore, the 2023 guidelines recommend against caffeine abstention for AF prevention [4].

Uncontrolled hypertension is a major risk factor for AF development, attributing one-fifth of AF cases to hypertension from the Atherosclerotic Risk in Communities (ARICs) study [47,48]. Data from the Framingham Heart Study similarly showed that hypertension doubled the 15-year risk of incident AF [49]. The SPRINT randomized clinical trial on patients with hypertension and elevated cardiovascular risk demonstrated that intensive blood pressure management lowered the risk of developing AF by 26% over a median follow-up of 3.8 years [50]. Long-term strict BP control in patients with AF reduces the risk of stroke, bleeding, and other cardiovascular outcomes, with each 5 mm Hg reduction lowering the risk of major cardiovascular events by approximately 10% [51,52]. Therefore, optimal hypertension control is strongly recommended in the management of AF [4]. Furthermore, renin–angiotensin system (RAS) blockers, including angiotensin-converting enzyme inhibitors and angiotensin II receptor blockers, have demonstrated their efficacy as anti-hypertensive agents in preventing recurrent AF, especially in patients with heart failure or left hypertrophy. A meta-analysis by Zhang et al. demonstrated a significantly lower risk of AF burden in RAS blocker users with an odds ratio of 0.65 [53]. The proposed mechanisms underlying their antiarrhythmic effects include a reduction in atrial fibrosis, structural and electrical remodeling, anti-inflammatory effects, and modulation of sympathetic nervous system activity [4,54,55]. In patients with hypertension undergoing catheter ablation, the ERADICATE-AF trial found that adding renal denervation to pulmonary vein isolation significantly improved the freedom from AF at 12 months (hazard ratio of 0.57) [56]. However, the SMAC-AF trial did not reveal any benefits from singularly aggressive BP management in the prevention of atrial arrhythmia recurrence post-catheter ablation for AF [57].

Sleep-disordered breathing (SDB) is highly prevalent in patients with AF, observed from 20% to 50% across different types of AF [58,59]. The VARIOSA-AF observational study found that SBD severity, as evidenced by the respiratory disturbance index on a specific night, correlates directly to AF risk during the same day with a 1.7-fold, 2.3-fold, and 10.2-fold increased risk for at least 5 min, 1 h, and 12 h of AF, respectively [60]. While several observational studies suggest that SDB treatment reduced the AF burden and AF recurrence after catheter ablation, randomized clinical trials have not consistently demonstrated the benefits of SDB therapy in reducing the AF burden or recurrence post-cardioversion [61,62,63,64,65]. Recent studies have emphasized the significant role of sleep hygiene on the management and prognosis of AF. A UK Biobank study involving 403,187 participants revealed that individuals with healthy sleep patterns had a 29% lower risk of developing AF, independent of traditional risk factors, compared to those with poor sleep hygiene [66]. Similarly, another retrospective study found that unhealthy sleep patterns significantly increased the risk of AF recurrence following catheter ablation, with a hazard ratio of 3.47. Conversely, adherence to a healthy sleep pattern was associated with a lower risk of AF recurrence post-ablation [67].

Beyond lifestyle modifications and optimization of cardiovascular risk factors, addressing non-cardiovascular comorbidities also plays a significant role in the management and prognosis of patients with AF [68]. For example, uncontrolled musculoskeletal conditions, such as osteoarthritis, have been reported to significantly impair the quality of life in older adults with AF [69]. Similarly, the presence of diabetes mellitus in patients with AF is independently associated with worse health outcomes, including a higher risk of major cardiovascular events, cardiovascular mortality, reduced quality of life, and increased healthcare resource utilization [70]. Optimal glycemic control in diabetic patients undergoing AF treatment, such as catheter ablation, may reduce the risk of AF recurrence [4,71]. Furthermore, recent findings from the Fushimi AF Registry showed that non-cardiovascular diseases, such as anemia and kidney or lung disease, were independent predictors of adverse outcomes in patients with AF, highlighting the importance of incorporating these non-cardiovascular comorbidities into individualized risk stratification and management in patients with AF [68].

### 3.2. Prevention of Thromboembolism

Several clinical risk scores have been developed to assess stroke risk in patients with AF, with each offering unique advantages and considerations for clinical use. Introduced in 2001, the CHADS_2_ score, which includes congestive heart failure, hypertension, age, diabetes, and history of stroke or transient ischemic attack, represents an advancement over previous classification systems and guides the selection of antithrombotic therapy [72]. Developed in 2010, the CHA_2_DS_2_-VASc score improved the prediction of thromboembolism (TE) by incorporating additional risk factors, such as vascular disease, female sex, and age categories [73]. The 2013 ATRIA score further refined age categories and included renal function and proteinuria, which helped classify more patients as either low or high risk [74]. The 2016 MCHA2DS2-VASc score, specifically designed for Asian populations, adjusts the lower age threshold and has proven more effective than CHA_2_DS_2_-VASc in identifying Asian patients with AF who could benefit from stroke prevention [75]. The GARFIELD-AF tool, launched in 2017, uses web-based data to simultaneously evaluate the risk of stroke, mortality, and bleeding, facilitating shared decision-making about the benefits and risks of anticoagulation [76]. Recently, the role of the sex component in the CHA_2_DS_2_-VASc score has been re-evaluated. Earlier studies found that females with AF had a significantly higher risk of ischemic stroke compared to men and were less likely to receive oral anticoagulation (OAC), resulting in more severe stroke outcomes for females [77,78]. Furthermore, the elevated risk in women was primarily observed in those over 65 or with at least one non-sex-related stroke risk factor [79]. However, the 2024 European Society of Cardiology guidelines for managing atrial fibrillation excluded female sex from the CHA_2_DS_2_-VASc algorithm, citing that it serves as an age-dependent stroke risk modifier rather than an independent risk factor [80].

The CHA_2_DS_2_-VASc score, while widely validated and outperforming the earlier CHADS_2_ score, has varying predictive accuracy across different populations. For example, the annual stroke risk for patients with a CHA_2_DS_2_-VASc score of 2 can range from less than 1% to over 2%, depending on the study [81]. For those with a CHA_2_DS_2_-VASc score of 1, anticoagulation decisions require a more individualized approach due to the score’s limited predictive ability [82]. Additional factors such as AF burden, blood pressure control, certain biomarkers (i.e., pro b-type natriuretic peptide), and the structure and function of the left atrium or its appendage can also significantly affect stroke risks [83]. In populations where CHA_2_DS_2_-VASc underperforms, such as renal disease, alternative scores such as ATRIA and GARFIELD-AF offer modest improvements in risk assessment, though their validation has been less rigorous [76]. The risk of bleeding must be considered with anticoagulation therapy for stroke prevention. However, several bleeding risk scores, including HAS-BLED, HEMORR2HAGES, and ATRIA, are unable to accurately categorize patients into high- and low-risk groups for bleeding because they include factors that also increase the risks of both stroke and bleeding [4].

When anticoagulation is indicated, direct oral anticoagulants (DOACs) are recommended over warfarin, except in patients with moderate-to-severe mitral stenosis, a mechanical heart valve, or specific thrombophilia (such as antiphospholipid syndrome), where warfarin remains the recommended treatment [4,84]. Furthermore, careful consideration should be given to switching from warfarin to DOAC in frail older patients with AF, as this approach has been associated with an elevated risk of bleeding complications without significant reduction in thromboembolic complications [85]. In patients with AF who have undergone percutaneous coronary intervention for acute coronary syndrome (ACS), dual antithrombotic therapy (OAC plus single antiplatelet therapy) lowered bleeding risks without compromising cardiovascular outcomes when compared to triple antithrombotic therapy (OAC plus dual antiplatelet therapy) [86]. The ESC guidelines recommend a short-term (one-week) triple therapy regimen in specific high-risk ACS subsets, such as ST elevation myocardial infarction, prior stent thrombosis, complex coronary procedures, and prolonged cardiac instability with an intermediate-to-low bleeding risk [80,87]. Furthermore, for ACS patients with diabetes who require anticoagulation, extending the triple antithrombotic therapy for 1–3 months may be considered when the risk of thrombosis is greater than the risk of bleeding [88]. The post-hoc analysis of the AFIRE trial with 2215 patients with AF and stable CAD over a median follow-up of 2 years found that rivaroxaban monotherapy is non-inferior to combination therapy with antiplatelet to prevent the composite endpoint of stroke, systemic embolization, MI, the need for revascularization, or death from any cause, while it is also associated with significantly less bleeding [89,90]. For AF patients unable to tolerate anticoagulants, left atrial appendage (LAA) occlusion (LAAO) has emerged as an alternative to oral anticoagulation for stroke prevention [4]. The PROTECT AF and PREVAIL trials demonstrated that the Watchman device is non-inferior to warfarin for the combined safety and efficacy composite endpoints (thromboembolism, cardiovascular death, clinically relevant bleeding, or procedure-/device-related complications) among patients with AF and CHADS_2_ scores ≥1 and ≥2, respectively [91,92]. Similarly, the PRAGUE-17 trial investigated 402 high-risk patients with AF at high risk for stroke and bleeding (mean CHA_2_DS_2_-VASc 4.7 ± 1.5) and showed that percutaneous LAAO was comparable to DOAC in preventing major AF-related cardiovascular, neurological, and bleeding events over a duration of follow-up of up to 30 months [93]. The OPTION trial further demonstrated that LAAO in patients who underwent catheter ablation for AF was associated with a reduced risk of major or clinically relevant non-major bleeding at 36 months and was non-inferior in the composite outcome of death from any cause, stroke, or systemic embolism [94]. Based on this evidence, the 2023 guidelines have upgraded the recommendation for percutaneous LAAO to a Class 2A for AF patients with a CHA_2_DS_2_-VASc score of 2 or higher and a contraindication to long-term anticoagulation [4].

### 3.3. Rate vs. Rhythm Control

The management of AF typically involves either rate or rhythm control strategies. Commonly used medications for rate control include atrio-ventricular (AV) nodal blockers, such as beta-blockers or calcium channel blockers (CCBs), though CCBs should be avoided in patients with left ventricular dysfunction. Digoxin can be added as adjunctive therapy, particularly for improving rate control in HF patients [4]. The 2010 RACE II trial demonstrated that lenient rate control (below 110 BPM) provides comparable outcomes for strict rate control (below 80 BPM) in patients with permanent AF, though stricter control may be reasonable for symptomatic cases [95]. A rhythm control strategy, on the other hand, aims to restore and maintain sinus rhythm through cardioversion, antiarrhythmic drugs, or catheter ablation procedures [4]. Previous studies, including the 2002 AFFIRM and the 2004 HOT CAFE trials, have shown that rate control has similar outcomes to rhythm control for all-cause mortality, thromboembolism, and major bleeding, with a lower risk of adverse drug reactions [96,97]. However, these trials were confounded by the toxic effects of antiarrhythmic drugs and the suboptimal treatment of risk factors and comorbid conditions. More contemporary studies have demonstrated that rhythm control may offer greater advantages for specific patient populations, including individuals with HF, symptomatic or recent-onset AF (within one year), younger patients, and those with fewer comorbidities [4].

- Rhythm control strategy

Electrical cardioversion is the preferred strategy for acute rhythm control in AF due to its greater efficacy and rapidity compared to pharmacological cardioversion alone [4,98,99]. Immediate synchronized electrical cardioversion is indicated for AF with associated hemodynamic instability, while pharmacological cardioversion can be an option for stable patients [98,100]. In patients with AF lasting ≥48 h, at least 3 weeks of therapeutic anticoagulation or imaging to rule out intracardiac thrombus is recommended before elective cardioversion, with continued anticoagulation for at least 4 weeks post-cardioversion to prevent thromboembolism. For those with LAA thrombus on imaging, anticoagulation should continue for 3–6 weeks before re-evaluation for cardioversion [4,101,102].

Flecainide and propafenone are effective for both the conversion of AF to sinus rhythm and the maintenance of sinus rhythm but are contraindicated in structural heart disease or HF due to the risk of proarrhythmia [4,103]. A beta-blocker or nondihydropyridine calcium channel blocker is generally given at least 30 min prior to prevent 1:1 AV conduction during atrial flutter [104]. For patients with recurrent AF outside a hospital setting, the “pill-in-the-pocket” approach, using a single oral dose of flecainide or propafenone with an AV nodal blocker, is an effective rhythm control strategy, provided it has been tested in a monitored setting. This approach allows for a rapid conversion to sinus rhythm without the need for daily medication [105]. Ibutilide is another pharmacological option for the cardioversion of AF with a rapid-acting onset (within 30–90 min), with conversion rates of about 30%, but it should be avoided in patients with LVEF ≤ 40% due to its independent risk of QT prolongation and torsade de pointes [106,107]. Alternative pharmacological options for AF cardioversion include intravenous amiodarone, which has a slower conversion time of up to 8–12 h, and intravenous procainamide, which is less effective and carries a relatively high risk of hypotension and HFrEF exacerbation [4,108].

For long-term maintenance of sinus rhythm, dronedarone is suitable for patients without recent decompensated HF or severe LV dysfunction. Dofetilide and sotalol are reasonable for long-term maintenance therapy for patients without a significant prolonged baseline QT interval, but they require QT interval monitoring due to the risk of torsade de pointes. Sotalol should be avoided in patients with HFrEF due to potential intolerance when combined with beta-blockers [4,109]. Amiodarone, though more effective than sotalol and Class IC agents, is associated with significant side effects, including pulmonary toxicity, thyroid dysfunction, and liver toxicity, making it a second-line option when other agents are ineffective or contraindicated. Amiodarone is also a preferred option for maintaining sinus rhythm for patients with HFrEF where most other drugs are contraindicated [4,109,110,111].

For patients on long-term amiodarone therapy, regular monitoring is essential to detect and manage potential adverse effects. Recommended assessments include thyroid function tests with thyroid-stimulating hormone (TSH) at baseline with free T4 and T3 if TSH is abnormal, at least once every 6 months, as well as ALT and AST on a similar schedule to detect hepatotoxicity. An annual ECG is advised to monitor for QT interval prolongation and arrhythmias. Due to amiodarone-associated pulmonary toxicity, primarily manifesting as interstitial lung disease or hypersensitivity syndrome with a 10% fatality rate, a baseline chest X-ray is recommended, along with further imaging if respiratory symptoms develop, to help identify potential lung complications. Pulmonary function tests, including carbon monoxide diffusing capacity, can be used to detect early changes in pulmonary function, although it is not routinely required [112]. Corneal microdeposits (epithelial keratopathy) are common with amiodarone use, and baseline ophthalmologic examination is not recommended but should be conducted if visual abnormalities or light sensitivity develop, indicating possible optic neuropathy [113]. Annual physical examinations are also necessary to check for dermatologic and neurological side effects, such as skin discoloration and peripheral neuropathy [4,110,111].

- Early rhythm control

Rhythm control for AF can be achieved through cardioversion (electrical or pharmacologic) for the acute management and long-term maintenance of sinus rhythm with antiarrhythmic drugs or catheter ablation [114]. Given the elevated risk of cardiovascular complications during the first year after an AF diagnosis, known as “early atrial fibrillation”, early rhythm control within this period has emerged as a beneficial strategy. The 2023 guidelines recommend a trial of rhythm control as a Class 1 indication for patients with reduced left ventricular function and high AF burden and as a Class 2a indication for those with recent diagnosis or symptomatic AF [4,115]. The EAST-AFNET 4 trial, which randomized 2789 patients with early AF and cardiovascular conditions over 5 years of follow-up, revealed that early rhythm control therapy reduced the composite outcome of cardiovascular death, stroke, or hospitalization for HF or acute coronary syndrome compared with rate control strategies (hazard ratio of 0.79) [116]. Subsequent subgroup analyses revealed that the benefit of early rhythm control was particularly pronounced in patients with a higher comorbidity burden (CHA_2_DS_2_-VASc score ≥ 4), with the composite outcome occurring in 23% of the early rhythm control group versus 34% in the usual care group (hazard ratio of 0.64) [117]. Similarly, several studies collectively confirmed the time-dependent effect of rhythm control initiation for AF management, with earlier intervention leading to better cardiovascular outcomes [118,119,120].

- Catheter ablation

Among rhythm control strategies, catheter ablation is more effective in patients with recurrent symptomatic AF despite antiarrhythmic drug therapy and in those without significant left atrial enlargement or multiple comorbidities [121]. Up to 90% of patients with paroxysmal AF achieved symptom-free 1-year post-ablation despite potential complications, such as thromboembolism (0.5–1% risk), tamponade, and vascular complications [122]. Therefore, catheter ablation has now received a Class 1 indication in the latest guidelines for rhythm control in certain patient populations [4].

**Figure 1:**
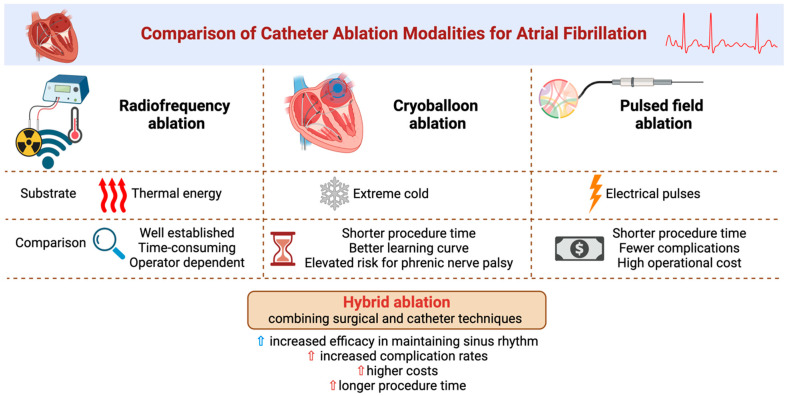
Comparison of catheter ablation modalities for atrial fibrillation.

Several AF ablation modalities now exist, including radiofrequency, cryoballoon, laser balloon, hot balloon, pulsed field, and hybrid ablation [123]. Radiofrequency ablation, a well-established, effective, and safe technique, uses thermal energy to ablate cardiac tissues. The recent introduction of contact force-sensing catheters optimized for temperature-controlled radiofrequency energy has further enhanced its performance, although it often requires longer procedures [124,125,126]. In contrast, cryoballoon ablation, which employs extreme cold temperatures, provides outcomes comparable to radiofrequency ablation while reducing procedural duration. Nevertheless, it carries a higher risk of phrenic nerve palsy [126,127,128]. Pulsed field ablation, a newer technique using electrical pulses to selectively target cardiac tissues, has shown promising efficacy and safety outcomes with shorter procedural times and fewer complications compared to cryoballoon ablation, though the high cost of its equipment can be a barrier [128,129]. Hybrid ablation, integrating both minimally invasive surgical and catheter-based techniques, has demonstrated superior efficacy in maintaining sinus rhythm compared to catheter ablation alone. However, this approach is associated with higher complication rates, higher costs, and prolonged procedure times [130,131] (Figure 1).

Pulmonary vein isolation (PVI) is currently the primary approach in catheter ablation for AF [123]. A meta-analysis comprised of six randomized clinical trials with 610 patients found that AF recurrence was significantly lower in patients receiving PVI over 1 year of follow-up compared with other ablation strategies (relative risk of 0.54) [132]. Future advancements in ablation strategies focus on improving outcomes by identifying patients unlikely to benefit from PVI through phenotyping and mapping for other AF triggers through randomized controlled trials on alternative ablation techniques following PVI failure [133].

AF and HF often coexist, with approximately 40% of HF cases being complicated by AF, leading to worse outcomes due to their bidirectional relationship and common risk factors [134,135]. Recent studies have shown that catheter ablation for AF in patients with HF with reduced ejection fraction (HFrEF) improves morbidity and mortality, resulting in guidelines recommending AF catheter ablation as a Class 1 indication in this group [4]. Furthermore, catheter ablation has been demonstrated to improve the quality of life and enhance cardiovascular outcomes in patients with AF and HFrEF who are already on guideline-directed medical therapy (GDMT). The 2018 CASTLE-AF trial, which studied 363 patients with symptomatic AF and LVEF < 35%, discovered that catheter ablation decreased HF hospitalizations and all-cause mortality compared to GDMT over 3 years of follow-up [136]. Subsequently, the 2023 CASTLE-HTx trial demonstrated similar benefits of catheter ablation compared with GDMT alone in preventing all-cause death and reducing the need for implantation of a left ventricular assist device or urgent heart transplantation among patients with symptomatic AF and end-stage HF [137]. Limited evidence exists for using catheter ablation for AF in patients with HF preserved ejection fraction (HFpEF). The 2019 CABANA sub-analysis in 778 patients with AF and clinically stable HF found that catheter ablation led to increased survival rates, quality of life, and decreased AF recurrence compared to GDMT, particularly in patients with HFpEF [138].

- Rhythm control and dementia

There is growing evidence supporting the association between AF and cognitive impairment and dementia, even in the absence of clinically overt previous stroke [139,140]. However, the underlying mechanism for this association is likely multifactorial, with uncertainty regarding whether shared pathophysiological processes or a direct causal pathway are the primary driving factors [141]. However, prior studies demonstrated that this association between AF and cognitive impairment/dementia persists even after adjusting for known risk factors and is even stronger in younger patients compared with older patients with a higher burden of shared risk factors [142,143]. Furthermore, the relationship also exhibits temporality with AF preceding cognitive decline and a biological gradient, with greater AF burden associated with worse cognitive impairment [142,144]. The potential pathophysiology of AF-induced cognitive dysfunction includes silent cerebral ischemia, transient cerebral hypoperfusion, and hypertensive events from fluctuations in cerebral blood flow, AF-related inflammation, microhemorrhage, and brain atrophy or systemic atherosclerotic vascular disease [145,146]. Furthermore, the impact of rhythm on cognitive outcomes in AF remains unclear. The EAST-AFNET 4 trial found no significant difference in cognitive function, assessed by the MoCA score at 2 years, between patients with and without rhythm control [116]. In contrast, an age- and sex-matched observational study demonstrated that AF ablation was associated with a lower risk of dementia over ≥3 years of follow-up [147]. Future randomized controlled trials focusing on neurocognitive function are warranted for this knowledge gap.

## 4. Atrial High-Rate Events

Atrial high-rate events (AHREs), also known as asymptomatic subclinical AF, have gained recognition due to the widespread use of implantable cardiac devices (CIEDs), ambulatory ECG monitors, and personal smart devices [148]. Current data indicate that AHREs occur in approximately 28.1% of patients with CIEDs, with the prevalence increasing with age and comorbidities such as hypertension and heart failure [149]. AHREs lasting more than 24 h are associated with a significantly increased stroke risk, which varies based on the CHA_2_DS_2_-VASc score [4]. A secondary analysis of the ASSERT trial revealed that AHREs exceeding 24 h are associated with an elevated risk of stroke or systemic embolism in patients > 65 years with hypertension, without prior AF, and with an adjusted hazard ratio of 3.24 [150]. Similarly, a retrospective study in patients with subclinical AF lasting over 24 h and a mean CHA2DS2-VASc score of 4.2 revealed a significant reduction in stroke risk with anticoagulation (hazard ratio of 0.28) [151]. The stroke risk associated with AHREs of intermediate duration (lasting between 6 min and 24 h) is lower than those with AHRE ≥ 24 h and may vary according to the CHA_2_DS_2_-VASc score. Brief episodes of subclinical AF generally carry a low risk of clinical events [152,153]. Based on level B nonrandomized evidence, the 2023 guidelines recommend oral anticoagulation to be considered with AHRE ≥ 24 h and a CHA_2_DS_2_-VASc score ≥ 2 and AHREs lasting 5 min to 24 h and a CHA_2_DS_2_-VASc score ≥ 3. This decision should be made within a shared decision-making framework that evaluates both the duration of the episode and the individual patient’s risk. However, those with an AHRE lasting less than 5 min and no other indications do not warrant anticoagulation therapy [4].

Recent trials have provided nuanced insights into the management of AHREs and subclinical AF in patients with CIEDs [149]. The NOAH-AFNET 6 trial found that edoxaban use did not significantly reduce the composite outcome of cardiovascular death, stroke, or systemic embolism among patients with AHREs detected by CIEDs. Furthermore, it was associated with a higher risk of death or major bleeding [154]. In contrast, the ARTESiA trial demonstrated that apixaban reduced the risk of stroke or systemic embolism compared to aspirin in patients with subclinical AF, with a thromboembolism rate of 0.78% versus 1.24% per patient–year, respectively [155]. A meta-analysis of these two trials confirmed that OAC with edoxaban or apixaban reduces the risk of stroke in patients with device-detected AF but increases the risk of major bleeding [156]. Interestingly, a sub-analysis of ARTESiA suggested that patients with subclinical AF may have a lower stroke risk than those with clinically diagnosed AF, raising the possibility that a modified and “less sensitive” CHA_2_DS_2_-VASc threshold could better identify high thromboembolic risk in this patient population [157]. These findings highlight the complexity of risk stratification and therapeutic decision-making in subclinical AF.

Recent advancements in artificial intelligence (AI), particularly deep learning models for automated ECG interpretation, have significantly improved the detection and management of atrial fibrillation, particularly asymptomatic and paroxysmal cases [158,159]. AI algorithms have shown promising results in identifying hidden AF in sinus rhythm ECGs and assessing AF burden, aiding in the identification of high-risk patients and guiding treatment decisions in embolic stroke of unknown source (ESUS) [160]. For example, one study using a convolutional neural network demonstrated that a single AI-enabled ECG could identify patients with AF during normal sinus rhythm with an overall accuracy up to 80% [161]. Further validation by Choi et al. and Rabinstein et al. reinforced the diagnostic performance of AI in detecting underlying AF in patients with embolic stroke of undetermined source [162,163]. Additionally, the integration of AI with wearable devices that use photoplethysmography data enables continuous heart rhythm monitoring, offering real-time, dynamic assessments of AF and stroke risk, which can facilitate timely interventions and personalized management [164].

## 5. Approach to Atrial Fibrillation for General Internists

A general internist’s approach to AF should focus on comprehensive risk factor modification, lifestyle interventions, and the adoption of a team-based care model with referrals to cardiology and cardiac electrophysiology. Primary care providers play a crucial role in initiating risk factor management and lifestyle interventions even before the development of AF, targeting obesity, physical inactivity, excessive alcohol consumption, smoking, diabetes, hypertension, and other comorbidities [4]. Early interventions addressing these risk factors can help reduce the risk of AF onset, its progression, and complications, including stroke [4]. An effective AF management should also involve close coordination led by general internists with multidisciplinary teams, including patients, their families, nurses, and specialists, to ensure personalized care tailored to the patient’s needs and local healthcare resources [165]. Randomized clinical trials and observational studies have shown that structured nurse-led follow-ups for AF improve treatment adherence and reduce hospitalization rates and cardiovascular mortality [166,167]. Furthermore, primary care providers should refer patients to cardiologists and electrophysiologists, especially patients with newly diagnosed AF and symptomatic AF, despite optimal rate control or medication intolerance, and those with underlying structural heart disease or HF, as they may benefit from early catheter ablation for rhythm control or surgical interventions [168,169].

## 6. Conclusions

The 2023 ACC/AHA/ACCP/HRS guidelines introduce significant advancements in the diagnosis and management of AF, emphasizing the importance of a patient-centered, holistic approach for both prevention and treatment. The shift toward a staged AF classification system, combined with a focus on lifestyle modifications and comprehensive risk factor management, highlights the necessity of early intervention in mitigating AF’s progression and associated complications. In addition, the guidelines also advocate for early rhythm control, especially with catheter ablation, in certain patient populations, including those with HF, symptomatic or recent-onset AF, younger patients, and those with fewer comorbidities.

## Figures and Tables

**Table 1:** **Lifestyle intervention and risk factor modification.** Key points from the 2023 ACC/AHA/ACCP/HRS guidelines for the diagnosis and management of AF.

Lifestyle Intervention	Key Findings	Clinical Impact on AF	Recommendations/Guidelines
**Obesity**	Direct atrial remodeling promoting AF progression. Every 5-unit BMI increase raises postoperative/post-ablation AF risk by ~10–13%. Weight reduction (≥10% loss) improves arrhythmia-free survival, while weight fluctuations increase AF recurrence.	Significant reduction in AF symptoms, recurrence, and burden with sustained weight loss.	Encourage weight reduction strategies for patients with BMI ≥ 27; maintain stable weight loss to prevent AF recurrence.
**Physical Activity**	Sedentary lifestyle increases AF risk. Aerobic exercise improves functional capacity, reduces AF burden, and enhances quality of life. Moderate-to-vigorous exercise (210 min/week) reduces and shortens AF episodes (ACTIVE-AF trial). Excessive endurance exercise (≥3 h/day) linked to increased AF risk in young athletes.	Moderate exercise lowers AF incidence and recurrence; excessive endurance training may increase AF risk.	Recommend regular moderate aerobic exercise; avoid extreme endurance training; ensure adequate rate control in active AF patients.
**Smoking**	Associated with increased AF incidence, recurrence, and worse outcomes (stroke, HF, death). Smoking reduces anticoagulation efficacy and increases stroke risk.	Smoking cessation lowers AF incidence, improves overall CVD outcomes, and may enhance anticoagulation effectiveness.	Strongly recommend smoking cessation through behavioral interventions and pharmacotherapy.
**Alcohol Consumption**	Alcohol intake (>3 drinks/week) linked to higher AF incidence, symptom burden, and progression. Reducing alcohol intake improves AF-related outcomes.	Lowering or abstaining from alcohol reduces AF risk and burden.	Advise alcohol reduction to ≤3 standard drinks/week or abstinence in patients at risk for AF.
**Caffeine**	Limited evidence that caffeine increases AF risk. Moderate caffeine intake may slightly reduce AF incidence.	Moderate intake does not adversely impact AF; may be neutral or mildly protective.	Routine caffeine abstention is not recommended solely for AF prevention.
**Hypertension Control**	Hypertension accounts for ~20% of AF cases. Intensive BP control reduces incident AF (SPRINT trial) and lowers major cardiovascular events. Renal denervation may improve AF-free survival post-ablation (ERADICATE-AF) but not confirmed universally (SMAC-AF).	Strict BP management reduces AF incidence, stroke, and CV events; may improve AF-free survival post-ablation.	Strongly recommend optimal BP control in AF patients; consider adjunctive therapies (e.g., renal denervation) on a case-by-case basis.
**Sleep-Disordered Breathing (SDB)**	SDB prevalent in 20–50% of AF patients. Severity correlates with increased AF risk (VARIOSA-AF study). Observational data suggest that SDB treatment reduces AF burden, but RCTs show mixed results.	Addressing SDB may reduce AF burden and recurrence, though definitive RCT evidence is variable.	Screen for and manage SDB in AF patients; consider CPAP or other treatments if indicated.
**Sleep Hygiene**	Healthier sleep patterns lower AF incidence by 29% (UK Biobank). Unhealthy sleep patterns increase AF recurrence post-ablation (HR = 3.47).	Good sleep hygiene lowers AF incidence and recurrence.	Assess and improve sleep quality as part of a comprehensive AF management strategy.

## Data Availability

All data used in this review article are from published content. Please see reference lists.
